# Correction to: Efficient DNA-assisted synthesis of trans-membrane gold nanowires

**DOI:** 10.1038/s41378-018-0012-7

**Published:** 2018-06-04

**Authors:** Maoxiang Guo, Iván Hernández-Neuta, Narayanan Madaboosi, Mats Nilsson, Wouter van der Wijngaart

**Affiliations:** 1 Department of Micro and Nanosystems, KTH Royal Institute of Technology, Osquldas väg 10, Stockholm, 100 44 Sweden; 2 Science for Life Laboratory, Department of Biochemistry and Biophysics, Stockholm University, Tomtebodavägen 23 A, Solna, SE-171 65 Sweden

Correction to: Microsystems & Nanoengineering (2018) **4**, 17084; 10.1038/micronano.2017.84; published online: 12 February 2018

Figure 2 and the descriptive text in the Section “SEM characterization of synthesized nanowires” of the previously published version of this Article were erroneous. The authors would like to replace Fig. 2 and section “SEM characterization of synthesized nanowires” with the figure and text below:

**Fig. 2:**
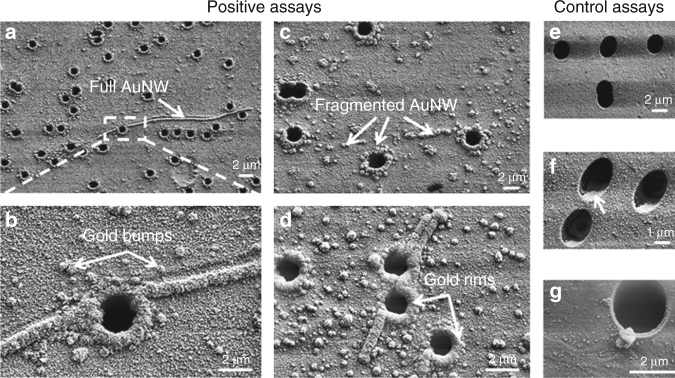
SEM images of membrane top surfaces after assays: **a**–**c** after a positive assay with 1 nM PLP; **d** after a positive assay with 50 fM PLP; **e** after a control assay with zero PLP concentration in which AuNPs incubation was omitted; **f** after a control assay with zero PLP concentration; and **g** after a control assay with 1 nM PLP concentration in which DNA stretching was omitted. In **a**–**c**, full AuNWs are visible; in **a**–**d**, gold rims and gold bumps are visible; in **c**, a fragmented AuNW is visible; in **b**–**d**, **f**–**g**, AuNPs are visible; in **f**, the arrow indicates a gold evaporation shadow. AuNPs, gold nanoparticles; AuNWs, gold Nanowires; PLP, padlock probes; SEM, scanning electron microscopy

## SEM characterization of synthesized nanowires

To study the AuNW formation, we captured SEM images of top membrane surfaces after assays (Fig. 2). For complete assays with PLP concentration ≥50 fM (Fig. 2a–d), we observed the following distinct features on the membrane surface: gold bumps with approximately spherical geometry, fragmented AuNWs (which are strings of gold bumps that stretch over the surface), full AuNWs that stretch over the surface and that have a visible length ranging from 3 to 60 μm, and gold rim structures around all pore openings. For control assays with zero PLP concentration (Fig. 2e, f), we did not observe any gold enhancement features. For assays with PLP concentration of 10 fM and for assays without RCP stretching (Fig. 2g), i.e., where drying of the RCP products occurred under natural convection instead of forced convection, we did observe gold bumps, but we did not observe full AuNWs, fragmented AuNWs, or gold rims. The size and areal distribution of the gold bumps and the full AuNWs are plotted in Fig. 3.

